# Innovation in regulation of rapidly changing health markets

**DOI:** 10.1186/1744-8603-10-53

**Published:** 2014-06-24

**Authors:** Gerald Bloom, Spencer Henson, David H Peters

**Affiliations:** 1 Institute of Development Studies, University of Sussex, Brighton, East Sussex BN1 9RE, UK; 2 University of Guelph, Guelph, Canada; 3 Department of International Health, Johns Hopkins Bloomberg School of Public Health, 615 North Wolfe Street, Baltimore, MD 21205, USA

## Abstract

The rapid evolution and spread of health markets across low and middle-income countries (LMICs) has contributed to a significant increase in the availability of health-related goods and services around the world. The support institutions needed to regulate these markets have lagged behind, with regulatory systems that are weak and under-resourced. This paper explores the key issues associated with regulation of health markets in LMICs, and the different goals of regulation, namely quality and safety of care, value for money, social agreement over fair access and financing, and accountability. Licensing, price controls, and other traditional approaches to the regulation of markets for health products and services have played an important role, but they have been of questionable effectiveness in ensuring safety and efficacy at the point of the user in LMICs. The paper proposes a health market systems conceptual framework, using the value chain for the production, distribution and retail of health goods and services, to examine regulation of health markets in the LMIC context. We conclude by exploring the changing context going forwards, laying out implications for future heath market regulation. We argue that the case for new approaches to the regulation of markets for health products and services in LMICs is compelling. Although traditional "command and control" approaches will have a place in the toolkit of regulators, a broader bundle of approaches is needed that is adapted to the national and market-level context of particular LMICs. The implication is that it is not possible to apply standard or single interventions across countries, as approaches proven to work well in one context will not necessarily work well elsewhere.

## Background

Over the past two or three decades there has been a rapid evolution and spread of health markets across low and middle-income countries (LMICs). By this we mean there is some form of financial exchange (inside and outside the legal framework) between the users and providers of these services in a large proportion of health care encounters. This has been associated with a significant increase in the availability of health-related goods and services in all but the most remote localities. Indeed, absolute shortages of primary care services and pharmaceuticals are no longer the prevalent issue in many countries, but instead the chief concerns relate to the safety and efficacy of health care and drugs, and costs that preclude access by the poor [1].

Given that health markets in many LMICs have evolved rapidly and with little or no planning, the development of support institutions has tended to lag behind. In many cases markets for health products and services are not well linked to the broader health system, and regulatory systems are weak and under-resourced. A significant proportion of transactions take place outside the legal framework. At an individual level, patients are subjected to unnecessary, dangerous and expensive treatments, whilst often not being referred for life-saving treatments when these are needed. A significant proportion of medicines are sub-standard or counterfeit [2] In addition, treatment-resistant organisms can develop due to inappropriate use of antibiotics, antivirals and anti-malarials, and the disconnection between health market actors and the rest of the health system diminishes the effectiveness of disease surveillance [3].

A rather narrow view of regulation is as a government function involving administrative and bureaucratic controls aimed at correcting market failures through laws, orders, and rules placed by government on enterprises, citizens, and government, itself [4]. This kind of government regulation plays an important role in protecting the public against incompetent medical practices and dangerous medicines. However, it has failed to live up to expectations in many countries because of the limited information available to the state on the functioning of markets, the limited capacity of the state to enforce regulations, and the potential for capture of the state by special interests or by its own rent-seeking officials. More generally, there is an increasing recognition that states, on their own, are unable to regulate the complex health systems of the 21st Century effectively. One possible implication is that states should withdraw from trying to regulate modern economies. However, a large body of evidence has shown that unregulated markets in health and many other sectors can lead to highly undesirable outcomes, particularly for the poor. This has led analysts to seek a deeper understanding of the relationships between public and private actors and how these relationships influence the degree to which markets meet social needs [5-7]. Alongside state regulation of enterprises (so-called public regulation), enterprises are seen to regulate one another ("private regulation") and even to regulate themselves through internal management arrangements ("self-regulation") [8]. Civil society organizations also play important regulatory roles. In the realm of public regulation, there is a recognised shift from ‘hard’ to ‘soft’ law [9], whereby ‘rules of conduct’ are applied, which have no legally-binding force but nevertheless influence behaviour [10]. There is also an increasing interest in regulatory partnerships between state and non-state actors.

Non-state actors have a long history of exerting regulatory powers. A variety of trade associations, such as the guilds in Medieval Europe, have long regulated suppliers of goods and services. Self-regulating professions have played a similar role. Commercial networks, including franchises, set and enforce standards by their members. It has long been recognised that there is a tension between the role of these associations and networks in regulating the technical competence and ethical behaviour of their members and in helping their members to improve their livelihoods, sometimes at the cost of the public good. The state and a variety of stakeholder groups, such as consumer associations and political movements, provide a countervailing influence to organised interest groups. The degree to which a regulatory framework meets social needs is largely an outcome of political competition between these stakeholders.

An important explanation of the need for regulatory arrangements in health and several other sectors is the asymmetry of information between the possessors of specialised knowledge and expertise and the rest of the population [11]. Societies have developed mechanisms to ensure that practitioners are competent and refrain from abusing the power this knowledge gives them. Associations of these experts, or organizations that employ them, are best placed to ensure the quality of their performance, but they may prioritise the interests of the suppliers of expertise. The state and other stakeholder groups tend to have less capacity to assess their expertise. This has influenced the outcome of the political competition described above. In addition, the understandings of "experts" are strongly influenced by their training and professional networks; they often ignore other perspectives, including those of the people they are trying to serve [12]. This can lead to inadequately informed policies, such as the attempts to regulate antibiotic use in a top-down fashion in contexts where a majority of the population seek care and medicines in informal markets, operating outside the formal and state-led regulatory system [13].

An effective regulatory arrangement needs to have social legitimacy so that transgressions are seen to be unethical. This can result in high levels of compliance without very heavy investment in policing of performance. The narrative that explains and legitimates the rules is important, since it contributes to the creation of social norms of behaviour. In countries, where access to health care is perceived to be a social entitlement, powerful participants in the health sector need to justify their behaviour in terms of the public good [14]. This may constrain the degree to which they can openly act in a self-interested manner. North argues that these internalised ethical rules of behaviour are an important pre-requisite to the development of the institutional arrangements to support a complex modern economy [15]. This aspect of a regulatory framework is an important element in the path dependency of regulatory arrangements.

Efforts by the governments of LMICs to import institutional arrangements for the regulation of health markets from the advanced market economies have had limited success [16,17]. In many cases, the underlying rule-making and enforcement systems are weak, and the lack of systems of accountability means that efforts to regulate can contribute to corruption rather than improvements in quality or access to health products and services [18]. New approaches are needed that build on existing arrangements in LMICs [19]. Thus, a number of authors have begun to explore options for the regulation of health markets in LMICs [20-22], emphasising the role of partnerships between the state, market actors, and civil society in the formulation and implementation of market governance arrangements. These arrangements must recognise and reflect the interests and incentives of market actors and address questions such as the following [18]. Why are the incentives for the provision of good quality health products and services weak? How can these incentives be augmented in the most effective and resource-efficient manner? And, how can the state and civil society organisations ensure that the health system takes into account public health needs?

This paper explores the regulation of health markets in LMICs. It outlines the objectives of regulation and identifies the targets of regulatory efforts. It then defines a conceptual framework for examining regulation of health markets in the LMIC context, lays out a range of options that can be packaged to enhance the efficacy and efficiency of regulation. Finally, it explores the context going forwards, laying out implications for future heath market regulation.

## Objectives of health market regulation

The following paragraphs explore the multiple objectives for regulating health markets and argue that different types of objectives can be addressed by different regulatory approaches. In regulating markets for health products and services, governments typically focus on a broad set of issues:

• Quality of care: Are providers of health services competent? Is health care safe and effective? Are medicines and medical equipment safe and effective?

• Value for money: Is health care available at a ‘reasonable’ price? Is it cost-effective? Is it affordable given the resources available to consumers of health products and services as well as society as a whole?

• Social agreement: Is health care seen to be provided in a fair and equitable way, in terms of both access and financing?

• Accountability: Is health care provided and paid for in a transparent way that holds key actors responsible?

Governments may also take into account issues of macro-economic growth and international trade by protecting local companies against competition and/or supporting these companies in foreign markets. They may also introduce regulations to influence market structure and increase competition. This paper does not address these issues and focuses on regulations specific to health, although we refer to some emerging challenges with regard to the latter issues in the final section.

Over time and across populations, expectations differ with respect to the performance of health markets and, by implication, what regulatory systems are expected to achieve. Key factors include the level of economic development, patterns of disease burden, complexity of health systems and access to information through the media and the Internet. The most basic aims of a health regulatory system concern the protection of the population against generally-recognised and high-level risks, for example dangerous and/or ineffective medicines, harmful practices by incompetent practitioners, the control of epidemics, and exposure to addictive drugs. The failure of government to provide this kind of basic protection, which can require considerable investment in infrastructure and institutional development, can challenge its very legitimacy.

As incomes rise, institutional and governance arrangements are strengthened, the health system becomes more sophisticated and expectations of citizens with respect to protection against risks tend to rise. Whilst the costs of achieving this can escalate rapidly, the existence of more complex institutional arrangements can make it possible for the health sector to provide products and services on the basis of trust between different providers, funders and users [23]. This implies that the perspective of government needs to shift towards the conditions needed in order for trust between market actors to be established and maintained. The creation of such institutional arrangements can be seen as the building of a social contract for health and health services [14].

## What and who is regulated?

In trying to fulfil the broad social objectives of health market regulation outlined above, governments have traditionally focused their efforts on health products and equipment, and practitioners and facilities engaged in the provision of health services (Table 1). For these, regulations have variously attempted to control the volume, safety and quality, and/or price [20].

**Table 1:** Regulation of health products and services

**Parameter**	**Health Practitioners/Providers**	**Medicines**	**Health facilities**	**Medical equipment**
Volume	Limits on numbers in medical school, residency, or licensed	Public procurement arrangements	Approvals to establish facilities	Limits on major equipment purchases
Safety and quality	Training and continuing education requirements	Product and/or process standards	Product and/or process standards	Product and/or process standards
		Licensing systems	Licensing systems	Licensing systems
		Product labelling requirements		
Price	Salary scales	Import restrictions	Control on service prices	Control on service prices
		Subsidies		
		Controls on product prices		

Adapted from Ensor and Weinzierl (2006).

The most common regulation of health markets in LMICs is the certification of health providers [24]. This is usually a mandatory requirement in terms of minimum educational conditions in order to practice for those with formal training as physicians, nurses and other health professions. In most LMICs, once the initial licensing standards have been met, there is little regulation that requires health providers to ensure that they maintain their skills.

The regulation of medicines and medical equipment in most LMICs has evolved and developed somewhat in recent years. Nearly all countries have regulatory agencies to register and monitor pharmaceutical safety, although these differ widely in their capacity and effectiveness. Indeed, many national drug authorities are ill-equipped to do testing for drug efficacy, safety and/or quality, whether for products manufactured domestically or imported. This situation is exacerbated by the weakness of controls on imports, especially in the case of informal trade from neighbouring countries. In most cases their ability to monitor for adverse events due to medicines is virtually non-existent.

Many LMICs have adopted essential medicines programmes whereby drug use in the public sector is restricted to a set of essential medicines. Most have difficulty applying these policies to the private sector, where a high proportion of drug sales take place in many countries. Weaknesses in controls on imports, as outlined above, provide an additional element of complexity.

The licensing of health facilities and equipment usually involves the development and application of physical standards with which compliance is required. In most LMICs these requirements are based on international standards. Whereas a number of LMICs have adopted standards for health facilities and equipment, most lack the capacity to undertake meaningful conformity assessment, including testing and inspection functions, and enforcement.

Beyond the safety and efficacy of health products and services, many LMIC governments make efforts to facilitate access by the poor. Such efforts most frequently take the form of price controls at the wholesale or retail levels of the supply chain or subsidies of an implicit or explicit kind at the point of supply. These controls are easier to achieve in the context of public sector provision, although weak administrative controls often mean that informal payments are imposed on users. Effective price controls are more difficult in the private sector due to weak enforcement capacity on the part of government and little incentive for compliance on the part of health product and service providers.

These approaches to the regulation of markets for health products and services have supported the creation of systems with the capacity to deliver safe and effective health services in some countries. However, in many others, people continue to face serious problems with the safety, effectiveness and cost of health services. This is associated with the limited reach of the formal regulatory system and the incentives that encourage practices that are not in the public interest. The disconnection between government aspirations to control the health system through administrative measures and reality is particularly apparent with efforts to regulate the private sector. Thus, while it may be plausible to stipulate the medicines that can be used for particular conditions in public hospitals and clinics, laying down systems of incentives and penalties to induce private providers to follow these rules is much more problematic. In most cases the revenues of private providers are dependent on the volumes of medicines they sell, and at the same time there may be pressure from patients for drugs to be prescribed even if they are not needed or are even harmful.

The nature of the value chains [25,26] for health-related products and services in many LMICs raises additional questions about the efficacy of administrative approaches to regulation. These can span the formal and informal sectors and involve both large and small enterprises, and often have weak linkages between different parts of the chain. The ultimate aim of the regulation of health products and services is to ensure they are safe, effective, and affordable at the point of use. The locus of much regulation is on inputs to the supply of these products and services; for example trained practitioners, manufacturers, or importers of drugs and medical equipment. The efficacy of such an approach is dependent, however, on the integrity of the value chain beyond the point of regulation and to the point of end-use. This will determine the extent to which the characteristics of the product or service are maintained beyond the point of regulation. For example, regulating the manufacture and distribution of drugs will be ineffective at ensuring safe, quality and efficacious products if there is an appreciable supply of unregulated and sub-standard imports and if retailers do not provide informed guidance about the use of these products.

The fact that the value chain for health products and services in many LMICs involves numerous formal and informal sector actors with weak linkages along the chain suggests that regulation needs to pay particular attention near to the point of use. However, it is easiest to regulate value chains at so-called ‘pinch points’ where there are a smaller number of critical actors [24,25]. These include, for example, manufacturers and importers of drugs as opposed to the multitude of informal market distributors. This suggests a need for the regulation of health products and services to be customised to fit local value chain contexts, and adapted as value chains develop and evolve over time. At the same time, regulation can be a driver of the restructuring of the value chain, for example through licencing arrangements that limit the number and/or characteristics of actors, with longer-term implications for wider regulatory approaches.

## Regulation of health products and services in LMICs

The regulation of health products and services needs to balance the costs and benefits incurred by the regulator, by actors along the value chain, by the eventual provider and by society, as a whole [20]. The combined costs should be less than the social costs of market failure that are being mitigated through the regulation. Regulators incur costs in developing and implementing the regulation and in undertaking conformity assessment and enforcement efforts. The costs of achieving compliance are borne along the value chain – the set of activities required to deliver a service or product to the market. In health markets, these include ingredient (input) suppliers, manufacturers, distributors, educational and training establishments, hospitals and clinics, etc. These costs include the upgrading of facilities and/or procedures, purchase of new equipment, training and establishing and maintaining administrative procedures. Both regulators and value chain actors in many LMICs lack these resources, such that while regulations may be efficient in principle, they are not implemented to the level where they are efficient. Alternatively, the relatively well off may use services that are regulated, while the poor use less expensive, unregulated services.

In order for the regulation to be effective, actors must be able to achieve compliance within the existing economic and technical constraints, and the regulation itself must be enforceable. From the perspective of the entities being regulated, this implies that the opportunity costs of compliance should be at least no more than the costs of non-compliance, including the direct costs of fines and sanctions and the loss of revenue or professional prestige from non-compliance. Regulators can enhance the costs of non-compliance through their enforcement actions (for example increasing the frequency of inspection) and the scale of penalties imposed when infractions are identified. However, regulator costs tend to rise in line with the scale and scope of enforcement. It is unsurprising, therefore, that regulators in LMICs lack the resources to implement enforcement regimes that achieve the desired rates of compliance [20].

Questions about the efficiency and effectiveness of administrative approaches to regulation are not restricted to LMICs. Indeed, a number of analysts of the advanced market economies have moved away from a state-centred understanding of regulation. So-called "decentred" understandings of regulation draw on five central notions [5]: (i) the complexity of interactions between actors or systems; (ii) the fragmentation of knowledge, power and control; (iii) the autonomy of actors and limited capacity to govern them; (iv) the level and nature of interactions and inter-dependencies between actors; and (v) the lack of a clear distinction between the public and private sectors. These understandings draw attention to the roles that a variety of state and non-state actors play in ensuring that the public interest is reflected in the operations of markets. This decentred understanding of regulation potentially provides a useful way forward in thinking about the future regulation of health products and services in LMICs.

**Figure 1:**
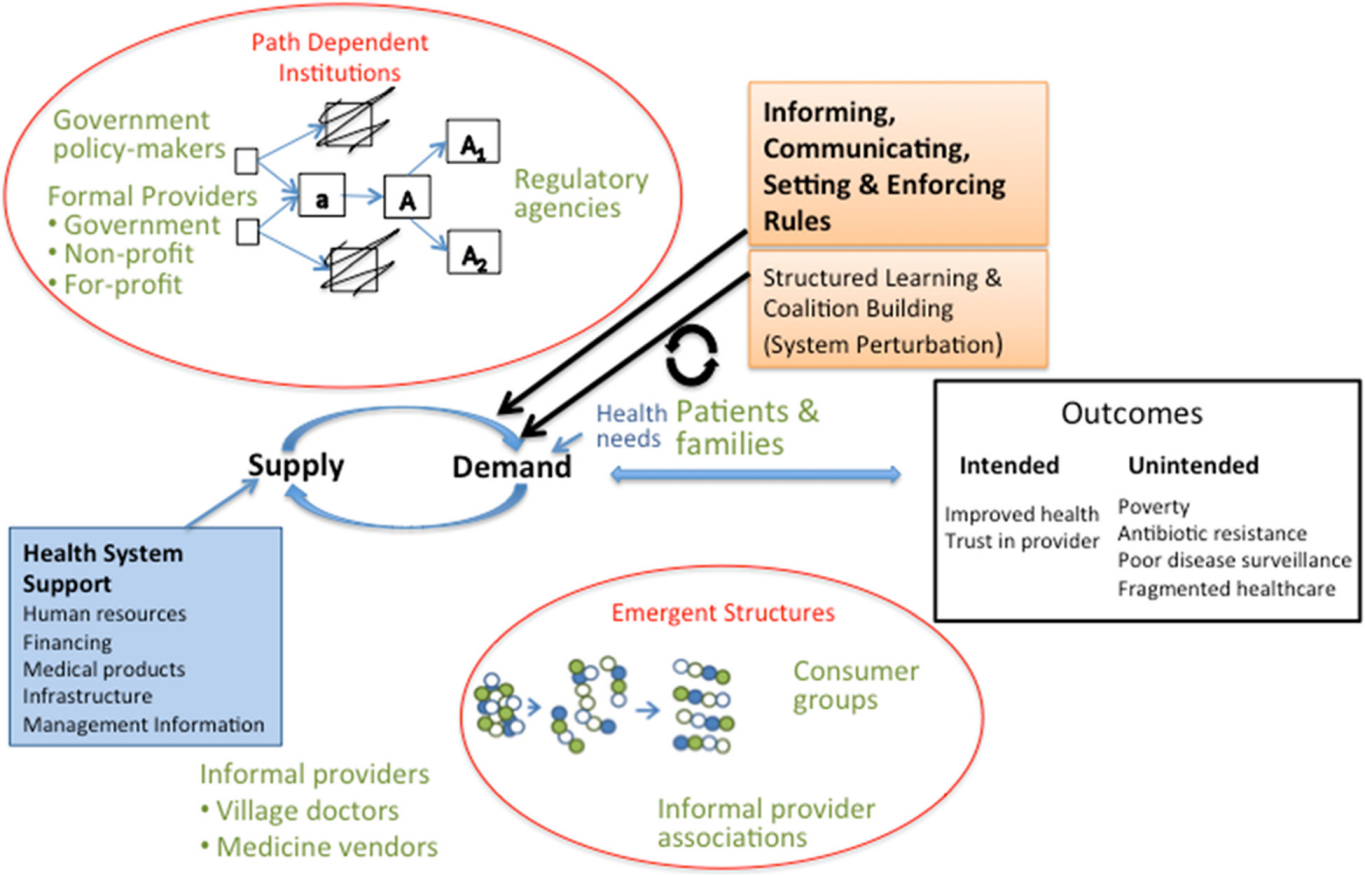
Health market systems and regulatory approaches.

Decentred understandings of regulation accord well with a market systems approach for assessing and defining alternative strategies for improving the performance of health markets, especially towards better meeting the needs of the poor [1]. Seeing health markets as complex adaptive systems enables us to explore options for regulation and the drivers of how these perform from a broader perspective than is the basis of administrative approaches (Figure 1). The supply and demand for health products and services is at the core of a health market system, and is influenced by regulatory efforts to inform, communicate, set and enforce rules. The model recognises that the demand for healthcare does not perfectly reflect health needs, due in part to information asymmetry, and knowledge, financial, geographic, and social barriers that impede demand.The market systems model suggests that a wide variety actors and institutions (shown in green in Figure 1) influence the performance of health markets, including both formally-recognised actors (for example doctors, hospitals and clinics, drug manufacturers, etc.) and informal actors and institutions (for example traditional healers, social norms and networks, etc.) that are inter-related and organised in varying ways. These actors rarely comply with a strict public-private dichotomy. Furthermore, the performance of the system needs to be considered not only in terms of the short-term delivery of health products and services, but also the long-term sustainability of the system. Account must be taken, therefore, of the scope for maintaining financial and human resource flows, upholding infrastructural and institutional capacities, and achieving sustained supplies of medical products and equipment (Health System Support in Figure 1).

Seeing health markets as complex adaptive systems suggests that the impacts of regulation need to be examined not only in terms of the supply and demand for products and services that comply with safety, efficacy, and affordability requirements, but also in terms of wider and often unintended and unanticipated outcomes [26]. For example, regulations can variously reinforce and undermine established market relations on the basis of trust, the net outcome of which is uncertain. They can also induce compensatory behaviours on the part of both providers and users of health products and services that can have wide-ranging consequences in terms of access of the poor to health products and services, drug resistance, ability to conduct disease surveillance, and structure of the healthcare system. A recent paper by Xiao et al., for example, illustrates how the introduction of a regulation aimed at reducing expenditure on pharmaceuticals in rural health facilities in China had differing impacts on health system performance between districts [27].

Complex systems also display path dependency, suggesting that regulatory institutions develop out of specific historical, economic and socio-political contexts, that are not simply reversible or replicable (illustrated in the top oval in Figure 1). For this reason, regulatory systems that work in one context may not work so well in another. Further, the emergence of regulation cannot be seen as distinct or divorced from the nature of the markets being regulated and the actors within the associated value chains. Thus, regulations tend to be co-constructed and driven by policy ‘entrepreneurs’ not only in government but also in commercial enterprises and other non-government entities [28]. At the same time, unofficial or social norms of market behaviour can alternatively precede or be induced by government regulation. An examination of the development of markets for specific products and services in advanced market economies has shown that the leading firms in a sector strongly influence the organisation of markets as an important element in their survival strategy [29]. These firms might lobby, for example, for the creation of standards that create barriers to entry by potential competitors. The outcome of this lobbying is strongly influenced, in turn, by the responses of other firms, other stakeholders and the state.The emergent properties of complex adaptive systems, in addition, mean that regulation can induce fundamental changes in the ways that both health markets and wider institutions are organised. For example, they may bring about processes of consolidation or proliferation at particular levels of the value chain, reinforcing or undermining value chain linkages, catalysing the self-organisation of value chain actors or users of health products and services, and empowering or disempowering regulatory officials and bodies. In Figure 1, this is shown as the organization of consumer groups and informal provider associations in the bottom oval. If and when these changes become reflected in new social norms, they will tend to be relatively robust, requiring quite profound changes to induce further processes of change. Of course, market actors recognise these processes and will make efforts to steer the course of regulation in their favour, as reflected in the notion of regulatory capture.

Theory and practice with complex systems, therefore, suggests that the design and implementation of effective and efficient regulation requires that the broad set of actors within markets for health products and services are brought together in processes of structured learning and coalition-building. This type of intervention is conceived as a perturbation to the market system that needs to continuously change in response to changes in the market (Figure 1). In so doing, the distinct and sometimes conflicting interests of these actors, their differing experiences and competencies, and prevailing power relations between them need to be recognised [3]. This is not easy to achieve in practice. As with any complex system, markets for health products and services are dynamic, requiring that different actors are involved in these processes over time and regulatory approaches are updated (or at least reassessed) on a continuous basis.

Recognition that regulations are co-constructed by regulators, health market actors and other non-government entities requires that political mechanisms be established that prevent undue influence by powerful interest groups. The performance of these mechanisms will reflect the degree to which the poor and relatively powerless are able to mobilise to ensure that their interests and points of view are taken into account. The outcome will involve the negotiation of rules that have wide social acceptance as legitimate and which define agreed moral norms of behaviour. Examples might include the widespread agreement that drugs need to be safe and reliably effective, that health workers should not prescribe dangerous drugs, and that very sick people should be referred to hospital and provided appropriate care. The negotiation of these norms is a political process that inevitably involves conflicts between different interests and understandings. However, such rules not only act to constrain the behaviour of market actors, but can also be critical preconditions and catalysts for effective linkages within value chains, acting to induce trust and reduce transaction costs.

The institutional arrangements for health-related markets in the advanced market economies were created over decades through quite gradual processes that reflected the path dependencies and emergent properties described above [30,31]. Regularised practices became established whereby broad norms of behaviour were established amongst market actors and regulators, with the expectation that transgressors would be punished. In turn, this meant that regulatory and enforcement resources could be used more expeditiously, with greater attention given to emerging issues and transgressions at the margin. These institutions were built upon a broader social consensus of what constitutes fairness and legitimacy of social arrangements – what has been called a "welfare regime" [32].

The situation in many LMICs today is very different, reflecting the rapid emergence and spread of health markets. In general, there have not been the opportunities for linkages and relationships between actors within health product and service value chains, and between these actors and regulators, to emerge and for behavioural norms to become established. Thus, the onus is on government regulation to moderate behaviour, with regulators looking to the advanced market economies for examples of ‘best practice’ that can be implemented ‘off the shelf’. A market systems perspective, of course, warns that regulatory approaches do not necessarily transfer well. What works well in health markets with limited types of formal sector actors and a relatively well-resourced regulator, may be ineffectual in the context of informal markets with a large variety of actors and an under-resourced regulator. This suggests that a special effort will be needed to facilitate the forging of new kinds of partnerships that can begin to create effective institutions to regulate these markets.

## Potential regulatory strategies

The foregoing discussion has highlighted how administrative approaches to the regulation of markets for health products and services may be ineffective and inefficient in LMICs. The shift in perspective towards decentred views of regulation and the understanding of health markets as complex adaptive systems suggests opportunities for regulating in innovative ways. At the same time, the nature of health market systems suggests that regulations are co-constructed by many actors and that these can have wide-ranging and sometimes unintended consequences that can have profound implications for the ways health markets are organised and operate. Defining alternatives to traditional regulatory modes needs to be approached with some care.

In approaching the implementation or reform of health market regulation in LMICs, it is important to recognise and build on established formal and informal rules and norms that influence the behaviour of health product and service providers and all actors along the value chain. It is also important to appreciate how efforts to implement more effective regulation relate to (and are dependent upon) other supply and demand-side interventions. For example, financial incentives may be linked to efforts to build and reform institutions and human capital, consumer education and empowerment, or wider policy reforms (such as in the area of trade, consumer product safety, and intellectual property). For many policy actors this requires a change in perspective and culture away from a preoccupation with established and formal institutions such as medical colleges, pharmacies and hospitals.

Table 2 presents a categorisation of potential strategies for the regulation of health product and service markets in LMIC, building on the work of others [20,21,33]. A distinction is made between administrative and bureaucratic controls (for example official registration and licensing systems), market supply-oriented approaches (for example self-regulation and contracting), consumer and/or citizen-oriented approaches (for example access to information), and collaboration-oriented approaches (for example co-production of products and/or services). Cutting across the options in Table 2 are interventions at various levels of the value chain, from training of practitioners and the manufacture of drugs and medical equipment through to end product and service markets.

**Table 2:** Regulatory strategies in health markets

**Regulatory strategy**	**Action**	**Weaknesses**
**Administrative and bureaucratic controls**		
Criminalisation of malpractice	Standards of practice are backed by criminal penalties	Complex and inflexible rules. Enforcement may be difficult, time-consuming, and costly. High compliance costs and the courts and regulators must be seen as independent.
Licensing and accreditation of providers and facilities	Standards based requirement to provide services or sell product applying to health facilities, health workers, or products	Needs information available to all actors. High costs of maintenance and enforcement for some items.
Product registration (e.g. drugs, vaccines, medical equipment and supplies)	Health products must meet specified standards. Often extends to requirements for importation or for labelling and advertising.	Costly and complex to enforce if testing is required. Needs high information and testing capabilities.
Product surveillance	Post-marketing	Expensive and potential for bias in collecting information. May be difficult to attribute health outcome to product.
**Market supply-oriented**		
Self-regulation	Association of providers or suppliers of goods and/or services sets standards which provide either a voluntary or enforceable code. Can be linked to a system of certification.	Requires government and public trust of providers. Danger of regulatory capture. Difficult to manage incentives collectively.
Contracting	Government purchases services from provider at verified quality, quantity, and/or price standards	Information gaps present. May have high administrative and technical requirements. Monopoly of providers may limit competition
Incentives and subsidies	Funds or other inducements provided for desired provider behaviour (e.g. location of practice, quality of service, permission for private practice, etc.)	Information gaps prevalent. May not prevent poor behaviour.
Disclosure	Offenders and poor performers are "named and shamed"	Requires assessment and communication seen as independent and trustworthy. Need viable alternatives for providers
Management improvement	Health providers (and organisations) trained and supported to improve quality and safety	Time consuming and potentially costly. May produce little change in incentives on its own -- a supportive strategy dependent on additional regulatory strategies.
**Consumer or citizen-oriented**		
Consumer education	Efforts to inform and educate consumers about the safety, quality and efficacy of health products and services and how to judge this at the point of provision	Difficult to reach and impact on most vulnerable consumers, namely the poor. Potentially very costly.
Right to information by citizens	Legal requirement to provide basic information.	Cost of collection and analysis of information and often difficult to enforce.
Consumer rights	Patient rights are identified and protected by law.	Places onus on individual to report violations that have already occurred. Need for possibly expensive system for arbitration.
Patient redress	Patients have ability to identify violations and seek resolution with provider organization or agreed arbitrator.	Places onus on individual to report violations that have already occurred.
Citizen empowerment	Communities or civil society organizations are provided with authority, resources, and capability to set local policy, assess performance, and sanction and reward.	Wide variation across communities in capabilities and interests; May be costly. Capture by local elites possible. May be hard to implement consistently on a large scale.
Liability norms	Definition of strict or liability standards that enable users of health products and services to sue for damages should injury occur.	Requires that citizens have access to the resources to pursue liability claims, or that class action is possible. Dependent on ability to relate cases of harm to specific health products or services.
**Collaboration oriented**		
Co-production (of services and regulation across key stakeholders)	Health providers, along with government agencies, private companies and/or consumer groups negotiate and share power, authority, and resources to ensure quality, safety, price or coverage of health services and products.	Honest broker may be needed to facilitate collaboration. Information gaps present. Need to continuously assess and renegotiate arrangements (is this a weakness?). Danger of capture by the powerful.
Partnerships for transparency and accountability	Government, civil society actors, providers, and/or independent technical experts set locally measurable and enforceable standards for performance.	May require external facilitation and convening. May address limited scale and scope of issues.

Collectively the four approaches in Table 2 suggest that the propensity of health markets in LMICs to deliver products and services that are inaccessibly priced and/or are substandard in terms of safety, quality and/or efficacy emanates from a number of constraints. First, and in many ways the overarching issue, are fundamental imperfections and asymmetries in access to information. Many of the options in Table 2 address this directly, for example through standard-setting, product registration and licensing. In part the weakness of information in health markets reflects the fact that perceptions of safety, quality and efficacy of health products and services reflect, at best actual experiences, and in many cases rely on poorly defined characteristics of suppliers that are believed to denote quality. The market failures in such contexts are well documented [33]. For example, there is a tendency for ‘bad’ products to crowd out ‘good’ products when the user is unable to distinguish adequately between these. Further, in the absence of reliable information on the safety and efficacy of products and services, users are driven to use proxies, such as price, which are imperfect at best and can be used as the basis of false product differentiation.

Second, the need for informed and empowered users of health products and services that drive competition in markets and the performance of providers on the basis of safety, quality and efficacy. In part this is dependent on users being informed – why safety, quality and efficacy matter in terms of the impacts of products and services on their health – and also being able to distinguish products and services according to these characteristics. The information imperfections described above are critical here. Requirements for the disclosure of information to consumers on health products and/or services, patient redress, and disclosure-based remedies are aimed directly at the empowerment of consumers.

The issue of trust is a critical issue in health product and service markets in LMICs. In the context of significant information asymmetries, relations along the value chain are dependent on trust if the potentially prohibitive transaction costs associated with verifying the safety, quality and efficacy of products and services are to be avoided. Trust-based value chain relations are especially problematic in the context of rapidly-evolving markets, as is typical of LMICs. Some of the options in Table 2 aim to act as proxies for trust or to offset high transaction costs. Examples include licensing and accreditation arrangements for health producers and facilities, codes of practice linked to certification, and the establishment of branded products and services, that are known for their quality.

Finally, there are concerns about the cost of implementing and maintaining effective regulation of health markets in LMICs. In many countries, the regulatory capabilities and the underlying institutional capacity are weak. Whereas capacity-building, perhaps backed by donor support, is one solution to this problem, the resource requirements can be prohibitive. Thus, a number of the options in Table 2 focus on self-regulation, incentives and subsidies, and management improvement.

Given the complexity of issues surrounding health markets in LMICs and also the nature of the value chains for health products and services, it is likely that a multi-pronged approach will be needed to improve the performance of markets for health-related goods and services. Also, reliance on a single regulatory measure is likely to induce compensatory or evasive behaviours on the part of market actors. We need to think about packages of complementary regulatory measures, the precise mix of which will be context specific. Taking drugs as an example, where value chains are well developed and have a high degree of integrity, most regulatory efforts can focus at the level of manufacture and/or importation, complemented by measures that control the right to prescribe many drugs. Where this is not the case, more intensive regulation and enforcement efforts are needed in distribution and end-product and service markets. The implication is that regulatory strategies will need to be defined and adapted according to local contexts, and adjusted as health markets develop and evolve. This recognises that processes of learning are inherent to health market systems, and also that regulations themselves are constituents of emergent processes that bring about broader changes to health markets and the associated value chains and wider institutions and infrastructure.

Regulation takes place at local, national, regional and international levels. There are potentially important roles for institutions at the regional and global levels, through the promulgation of regional or international standards and promotion of ‘good practice’ [34]. LMICs could make greater use of regulatory capacities beyond their borders as a means to reduce regulatory costs. It is important to recognise, however, that global standards are rarely promulgated with an eye to the specific context of informal health markets that predominate in many LMICs, and they may have little or no influence over the ways that health products and services are provided in practice. Further, whilst there are understandable incentives to use international private or non-governmental organization (NGO) providers of health products and services that are subject to more rigorous regulatory regimes in their home country, this does little to engender local regulatory or compliance capabilities.

Reforming approaches to the regulation of health markets in LMICs is not something that can be driven from outside. The ultimate aim has to be the establishment of rules that are recognised as legitimate by all stakeholders in the provision and use of health products and services, and that are internalised as behavioural norms. A recent paper by Ahmed et al. on Bangladesh suggests four key elements for what it calls "better management of pluralism" [35]: (i) participatory governance mechanisms, (ii) effective regulation and accountability; (iii) common information systems and (iv) building competencies for pluralistic governance. This will entail a process that involves a wide range of actors from both within and outside health markets. Health practitioners, manufacturers and/or suppliers of drugs and medical equipment, consumer representatives, policy-makers, researchers and the like clearly need to be ‘at the table’. But so also do political leaders, the media, faith groups and other elements of civil society. The outcome will be strongly influenced by the degree to which different social groups can mobilise to ensure that political leaders take their interests and perspectives into account.

## Conclusions: building regulatory institutions in a rapidly changing context

Many LMICs face the challenge of creating institutional arrangements for their health systems in a context of rapid change and rising public expectations. Attempts to import models from the advanced market economies are often not effective. These countries cannot retrace the lengthy process through which the latter countries created their regulatory arrangements. There is little systematic evidence on strategies for building effective institutional solutions to the problem of asymmetric information in low and middle-income countries [18]. Countries will have to pursue a learning-by-doing strategy, in which they test alternative interventions and build on what can be shown to work.

In building new regulatory arrangements, LMICs face several special challenges. The first is the degree to which market structures and norms of behaviour have become established in the informal and formal sectors in many countries. The process of change is likely to be highly contested and complex.

The second is role of the relatively small number of large multi-national companies that supply pharmaceuticals and diagnostic technologies. They have actively engaged in the creation of the regulatory frameworks in the advanced market economies. However, they have largely viewed LMICs as potential markets, without becoming strongly engaged in the creation of effective institutions. This has contributed to the very large market in counterfeit drugs and to the inappropriate use of many pharmaceuticals [2]. Experience over many years suggests that LMIC governments cannot address these problems alone; their capabilities and resources are simply inadequate. This suggests the need for explicit and transparent regulatory partnerships with multinationals and other stakeholders through the value chain that put in place the necessary controls whilst recognising the risk of regulatory capture.

The third is the emergence of new health-related companies in rapidly growing middle-income countries. Whereas the regulatory framework in the advanced market economies has restricted vertical integration between pharmaceutical companies, retail pharmacy chains, and prescribers of medications to reduce the incentive to sell excessive volumes of medicines, this may not be the case in many LMICs. It is not unreasonable to expect that complex ownership structures will emerge in these countries with a significant degree of vertical integration and horizontal market concentration. At present there are no agreed global regulatory standards relating to the ownership and vertical integration of health systems, putting the entire onus on national governments to put controls in place. Not only is it highly questionable that these governments have the required capacities, but there is a lack of horizon scanning at the global level to identify where, when and why problems occur and what can be done about them.

The fourth is the speed of potentially disruptive innovations in information and communication technologies (ICTs) and in point of treatment diagnostics. The rapid diffusion of access to the internet through mobile phones is enabling people in countries with weak health systems to gain access to expert advice and to products and services at more accessible prices [36]. However, mechanisms for ensuring the quality of information provided to consumers are weak, especially in LMICs. Further, these technologies have the potential to act as powerful new pathways for major stakeholders to establish large market shares. The rapid diffusion of ICTs in LMICs is also creating opportunities for the more effective and efficient regulation of markets for health products and services. Thus, we are observing examples of social networks in a number of LMICs, which enable consumers to distribute information on sub-standard products and/or service providers. These technologies alone, however, are not sufficient effectively to ‘discipline’ providers of products or services that are of poor quality. The overall impact of ICTs on health systems and the degree to which it makes them more accountable for quality and costs, will depend, to a large extent, on the regulatory framework that governments put in place.

The history of health system development in the advanced market economies has shown that decisions made early on can have profound effects for many years in the future. This suggests that the outcome of current efforts to build appropriate institutional arrangements for a modern health system in LMICs will have a powerful influence on future development. That is why it is particularly important that health system analysts understand the structure and operation of the complex markets that have emerged and build systematic knowledge on effective strategies for influencing their performance. The creation of appropriate institutional arrangements to regulate complex health markets will be an increasingly important health priority in coming years, and one to which all those with an interest in the access of the poor in LMICs to safe and effective health products and services must attend.

## Competing interests

The authors declare that they have no competing interests.

## Authors’ contributions

GB, SH and DP drafted sections of the paper and revised several drafts. All authors read and approved the final manuscript.
